# A New Canthinone-Type Alkaloid Isolated from *Ailanthus altissima* Swingle

**DOI:** 10.3390/molecules21050642

**Published:** 2016-05-16

**Authors:** Hye Mi Kim, Jin Su Lee, Jurdas Sezirahiga, Jaeyoung Kwon, Miran Jeong, Dongho Lee, Jung-Hye Choi, Dae Sik Jang

**Affiliations:** 1 College of Pharmacy, Kyung Hee University, Seoul 130-701, Korea; hyemi586@gmail.com (H.M.K.); jurdas30@gmail.com (J.S.); jchoi@khu.ac.kr (J.-H.C.); 2 Department of Life & Nanopharmaceutical Sciences, Kyung Hee University, Seoul 130-701, Korea; lee2649318@naver.com (J.S.L.); jeongmiran@hanmail.net (M.J.); 3 Department of Biosystems and Biotechnology, College of Life Sciences and Biotechnology, Korea University, Seoul 136-713, Korea; kjwkjy1207@gmail.com (J.K.); dongholee@korea.ac.kr (D.L.)

**Keywords:** *Ailanthus altissima*, simaroubaceae, canthinone type alkaloids, nitric oxide, inflammation

## Abstract

The present investigation of the chemical constituents of the stem barks of *Ailanthus altissima* has resulted in the isolation of six canthinone-type alkaloids, including a new compound, (*R*)-5-(1-hydroxyethyl)-canthine-6-one (**1**), and five known compounds (**2**–**6**). Moreover, four phenyl propanoids (**7**–**10**), two lignans (**11** and **12**), two triterpenoids (**13** and **14**) and a fatty acid (**15**) having previously known chemical structures were isolated during the same course of this study. The structure of the new compound was elucidated by physical (m.p., [α]_D_) and spectroscopic data (^1^H-NMR, ^13^C-NMR, 2D NMR, and HR-DART-MS) interpretation and its absolute configuration was determined by electronic circular dichroism (ECD) data and quantum chemical calculations. The inflammatory activities of the isolates were screened on lipopolysaccharide (LPS)-induced nitric oxide (NO), a proinflammatory mediator, in RAW 264.7 cells. Among these isolated compounds, six compounds exhibited significant inhibition of NO production, with IC_50_ values in the range of 5.92 ± 0.9 to 15.09 ± 1.8 μM.

## 1. Introduction

*Ailanthus altissima* Swingle (Simaroubaceae), the tree-of-heaven, has been used to treat diarrhea, dysentery, heat ailments, epilepsy, asthma, ophthalmic diseases, and it has also been used as an astringent. The stem bark of *A. altissima* has exhibited various biological activities, such as anti-proliferative, cytotoxic, anti-plasmodial, anti-malarial, anti-viral, antibacterial, anti-fungal, and analgesic activity [1,2]. Additionally, it was reported that a decoction of *A. altissima* decreased the production of inflammatory cytokines, TNF, IL-6 and IL-8 as well as NF-κB activation on the phorbol 12-myristate 13-acetate and calcium ionophore A23187 (PMACI)-stimulated human mast cell line, HMC-1 [3]. Furthermore, the EtOH extract of *A. altissima* inhibited the generation of the cyclooxygenase-2 (COX2)-dependent phases of prostaglandin D2 in bone marrow–derived mast cells (BMMC) [4]. Previous phytochemical investigations of *A. altissima* revealed the presence of alkaloids, terpenoids, steroids, and flavonoids [5]. Among these compounds, quassinoids and indole and β-carboline alkaloids are common major constituents of *A. altissima* [6,7,8]. Alkaloids from *A. altissima* are reported for their anti-herpes [9] and anti-mycotic properties [10] and for their action on the rate of intestinal blood flow in rabbits [11]. Indole and β-carboline alkaloids have shown inhibitory activity on cyclic adenosine monophosphate (cAMP) phosphodiesterase [12]. Moreover, it was demonstrated that canthin-6-one and its derivatives have anti-proliferative and cytotoxic activity [13], leishmanicidal activity [14], and gastro-protective effects [15]. Although most β-carboline and canthin-6-one alkaloids have been reported to show diverse pharmacological effects, there are few studies regarding their anti-inflammatory effects. 

**Figure 1:**
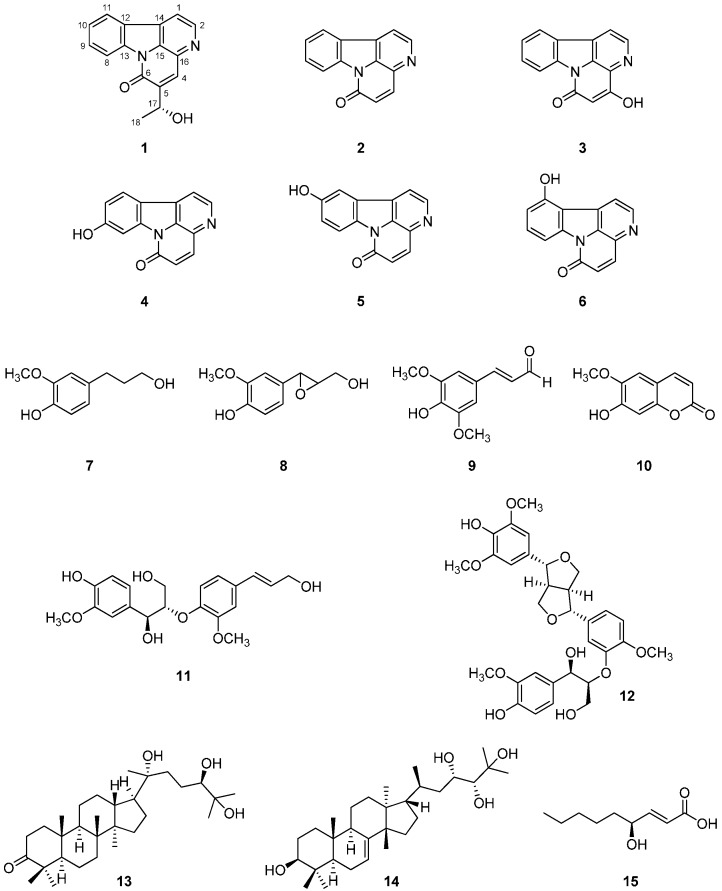
Structures of **1**–**15** isolated from the stem barks of *A*. *altissima.*

Recently it was reported that some quassinoids from the stem barks of *A. altissima* inhibited the production of nitric oxide in RAW 264.7 cells [16]. In our continuing study to search for anti-inflammatory agents from this plant, a new canthinone-type alkaloid (**1**), as well as 14 known compounds (**2**–**15**), was isolated further from the EtOAc- and BuOH-soluble fractions of the EtOH extract of the stem barks of *A. altissima* (Figure 1). Herein, this paper describes the isolation and structural elucidation of the isolates and their inhibitory effects on NO production in RAW 264.7 cells.

## 2. Results and Discussion

### 2.1. Identification of Isolated Compounds **1**–**15** from the Stem Bark of A. altissima

Compound **1** was isolated as a yellow amorphous powder, in which the molecular formula was established as C_16_H_13_N_2_O_2_ by HR-DART-MS (*m*/*z* 265.0992 [M + H]^+^; calcd. for C_16_H_13_N_2_O_2_, 265.0977) (Figure S8 in Supplementary Materials). The IR spectrum indicated the presence of a hydroxyl group (3159 cm^−1^), a conjugated carbonyl group, and an aromatic ring (1663, 1650, 1595 cm^−1^) (Figure S7). The UV spectrum of **1** displayed absorption bands at 229, 258, 298, 359, and 375 nm (Figure S6). It was concluded to be a canthin-6-one skeleton by comparing the ^1^H- and ^13^C-NMR data of **1** to those of **2** (canthin-6-one) [17]. The ^13^C-NMR spectrum of **1** (Table 1 and Figure S2) showed 16 carbon signals including a methyl, eight methines, and seven quaternary carbons. In the ^1^H-NMR spectrum (Table 1 and Figure S1), seven signals were displayed in the aromatic region. The *ortho*-coupled signals of the C ring at δ_H_ 8.16 (1H, d, *J* = 5.0 Hz, H-1) and δ_H_ 8.77 (1H, d, *J* = 5.0 Hz, H-2) and four mutually coupled aromatic protons at δ_H_ 8.60 (1H, d, *J* = 8.0 Hz, H-8), δ_H_ 7.74 (1H, dt, *J* = 8.0, 1.0 Hz, H-9), δ_H_ 7.58 (1H, dt, *J* = 8.0, 1.0 Hz, H-10), and δ_H_ 8.26 (1H, d, *J* = 8.0 Hz, H-11) were observed. The planar structure and ^1^H- and ^13^C-NMR chemical shifts were assigned by detailed analysis of 2D NMR spectra (Figures S3, S4 and S5), in particular HMQC, COSY, and HMBC. The ^1^H- and ^13^C-NMR of **1** exhibited strong similarity to those of **2**, except for the presence of the 1-hydroxyethyl group in the D ring. It was supported from δ_H_ 8.18 (1H, d, *J* = 1.0 Hz, H-4), which was long range–coupled with a H-17 at δ_H_ 5.16 (1H, dq, *J* = 6.5, 1.0 Hz), and δ_H_ 1.57 (3H, d, *J* = 6.5 Hz, H-18). Moreover, the ^13^C-NMR signals at δ_C_ 66.1 (C-17) and δ_C_ 23.4 (C-18) of **1** were shifted downfield due to the influence of the hydroxyl group.

**Figure 2:**
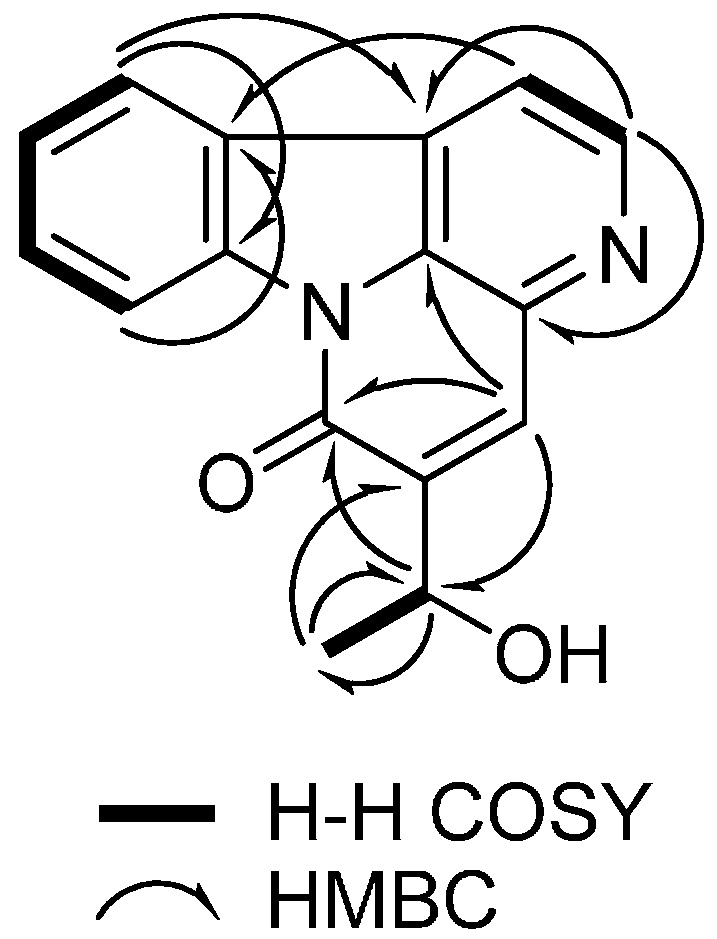
^1^H-^1^H COSY and HMBC correlations of **1**.

**Figure 3:**
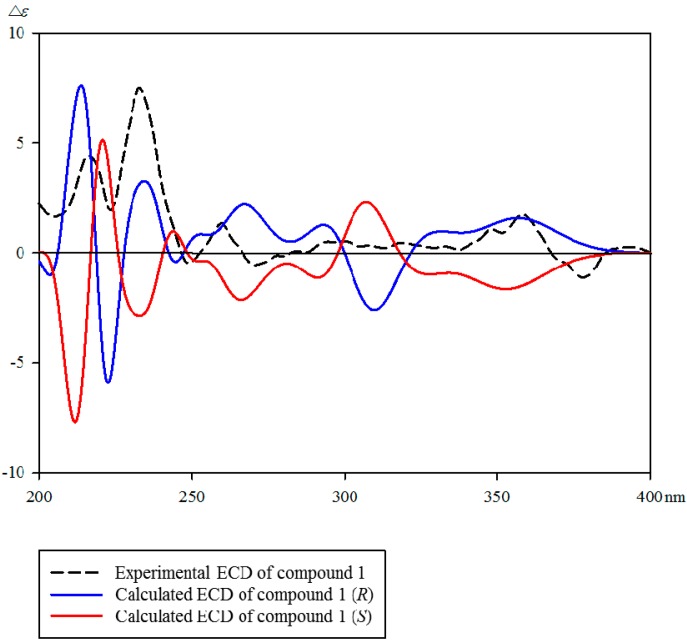
Experimental and calculated CD spectra of **1**.

Furthermore, the position of the 1-hydroxyethyl group was assigned by the observed HMBC correlations from H-4 (δ_H_ 8.18) to C-6 (δ_C_ 160.2)/C-17 (δ_C_ 66.1), H-17 (δ_H_ 5.16) to C-4 (δ_C_ 133.5)/C-6 (δ_C_ 160.2), and C-18 (δ_H_ 1.57) to C-5 (δ_C_ 147.8) (Figure 2). The absolute configuration of **1** was determined by comparing the experimental and calculated CD spectra using the time-dependent density functional theory (TDDFT) method. In the experimental electronic circular dichroism (ECD) spectrum of **1**, the positive cotton effect (CE) is observed at 215 and 230 nm. As shown in Figure 3, the calculated ECD spectrum of **1** exhibited strong positive CE around 210 and 232 nm, similar to the experimental spectrum of **1**. As a result, the absolute configuration of the hydroxyl group in **1** was determined to have an *R*-configuration. Therefore, the structure of **1** was determined as (*R*)-5-(1-hydroxyethyl)-canthin-6-one.

The structures of known compounds **2**–**15** were identified as canthin-6-one (**2**) [17], 4-hydroxycanthin-6-one (**3**) [18], 9-hydroxycanthin-6-one (**4**) [19], 10-hydroxycanthin-6-one (**5**) [20], 11-hydroxycanthin-6-one (**6**) [21], dihydroconiferyl alcohol (**7**) [22], epoxyconiferyl alcohol (**8**) [23], sinapaldehyde (**9**) [24], scopoletin (**10**) [25], *erythro*-guaiacylglycerol-β-*O*-4′-coniferyl ether (**11**) [26], ficusesquilignan B (**12**) [27], 20(*R*)-24, 25-trihydroxy-dammaran-3-one (**13**) [28], hispidol B (**14**) [29], and *trans*-4(*R*)-hydroxy-2-nonenoic acid (**15**) [30] by physical (m.p., [α]_D_) and spectroscopic data (^1^H-NMR, ^13^C-NMR, 2D NMR, and MS) measurements and by comparison with published values. Although 4-hydroxycanthin-6-one (**3**), 9-hydroxycanthin-6-one (**4**), 10-hydroxycanthin-6-one (**5**), and 11-hydroxycanthin-6-one (**6**) were isolated from the Simaroubaceae family, they have not been reported from *A. altissima*. Also, dihydroconiferyl alcohol (**7**), epoxyconiferyl alcohol (**8**), sinapaldehyde (**9**), and ficusesquilignan B (**12**) were isolated from this plant for the first time.

### 2.2. Anti-Inflammatory Activity of Isolated Compounds **1**–**15** from the Stem Bark of A. altissima

**Figure 4:**
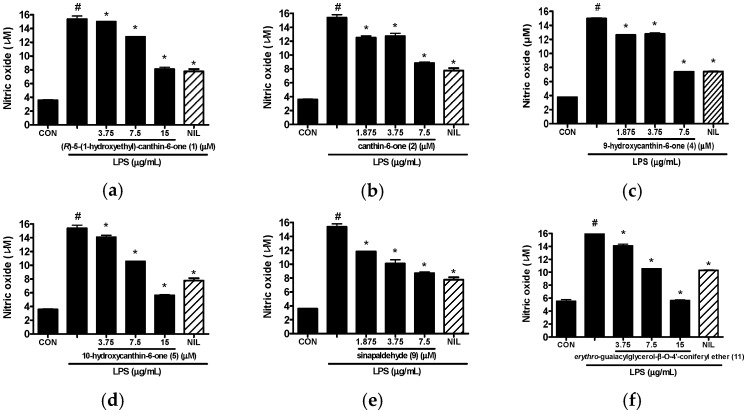
Effects of compounds **1** (**a**), **2** (**b**), **4** (**c**), **5** (**d**), **9** (**e**) and **11** (**f**) isolated from the stem barks of *A. altissima* on LPS-induced NO production in RAW 264.7 cells. Based on MTT (3-(4,5-dimethylthiazol-2-yl)-2,5-diphenyltetrazolium bromide) assay data, concentrations of isolates that would not affect cell viability were used for the following experiments. Cells were pretreated with the indicated concentrations of isolates for 1 h and then stimulated with LPS (1 μg/mL) for 24 h. NO level in culture media was measured by Griess assay. NIL (iNOS inhibitor, 10 μM) was used as a positive control to inhibit NO production. Values represent the means ± SD of three independent experiments. # *p* < 0.05 *vs.* the control group; * *p* < 0.05 *vs.* LPS-stimulated group. CON, control; NIL, l-*N*^6^-(1-iminoethyl) lysine.

All the isolates **1**–**15** from the stem bark of *A. altissima* were evaluated for their inhibitory effects on LPS-induced NO production in RAW 264.7 cells at non-toxic concentrations. As shown Table 2 and Figure 4, six compounds showed potent inhibitory effects on NO production (IC_50_ values ≤ 50 μM) and were assessed using IC_50_ values. Among the six canthin-6-one alkaloids isolated, 11-hydroxycanthin-6-one (**6**) was inactive in this assay system, while (*R*)-5-(1-hydroxyethyl)-canthin-6-one (**1**), canthin-6-one (**2**), 9-hydroxycanthin-6-one (**4**) and 10-hydroxycanthin-6-one (**5**) exhibited inhibitory activity with IC_50_ values ranging from 7.73 to 15.09 μM. The compound 9-Hydroxycanthin-6-one (**4**) showed more a potent inhibitory effect than canthin-6-one (**2**, IC_50_ value = 9.09 μM) with an IC_50_ value of 7.73 μM, indicating the hydroxylation at position 9 seems to increase the inhibitory activity. However, canthin-6-one (**2**) showed a more potent inhibitory effect with an IC_50_ value of 9.09 μM than (*R*)-5-(1-hydroxyethyl)-canthin-6-one (**1**, IC_50_ value = 15.09 μM), which has a 1-hydroxyethyl group at C-5. It was also observed that other canthin-6-one alkaloids possessing an additional hydroxyl group (**5** and **6**) exhibited weaker inhibitory effects than canthin-6-one (**2**). It seems that a hydroxyl group in canthin-6-one weakens the inhibitory effect with the exception of a hydroxyl group at C-9. However a further study is needed to ensure a structure-activity relationship for the canthin-6-one alkaloids. In a previously study, 9-hydroxycanthin-6-one (**4**) was reported to inhibit the NF-κB pathway in TNF-a–stimulated HEK-293/NF-kB-luc cells [31]. Among the isolates, sinapaldehyde (**9**) was found to have the most potent inhibitory effect with an observed IC_50_ value of 5.92 μM, and *erythro*-guaiacylglycerol-β-*O*-4′-coniferyl ether (**11**) also showed a significant inhibitory effect with an observed IC_50_ value of 10.69 μM. By comparison with our previous study, two methoxy groups at C-3 and C-5 might be the bioactive groups for sinapaldehyde (**9**) [16]. Morever, sinapaldehyde (**9**) has shown significant inhibitory activity on TNF-α–induced NF-κB transcriptional activity in HepG2 cells, ascribing the symmetrical methoxyl group a more important role in NF-κB inhibition [32]. However, the roles of these functional groups in the activity are not clear due to a limited number of isolates in the present study.

## 3. Materials and Methods 

### 3.1. General Procedures

Optical rotations were determined with a JASCO P-2000 polarimeter (Jasco Inc., Easton, MD, USA). UV and CD spectra were obtained on Spectramax M5 (Molecular Devices, Sunnyvale, CA, USA) and JASCO J-1100 spectrometers (Jasco Inc., Easton, MD, USA), respectively. IR spectra were recorded using an Agilent Cary 630 FT/IR spectrophotometer (Agilent Technologies Inc, Santa Clara, CA, USA). NMR experiments were conducted on a Varian 500 MHz, and the chemical shifts were referenced to the residual solvent signals. Chemical shift are presented in ppm. A mass spectrometer was an AccuTOF-TLC single-reflectron time-of-flight mass spectrometer equipped with a DART-SVP ion source (IonSense, Saugus, MA, USA). TLC analysis was performed on Kieselgel 60 F_254_ (silica gel, 0.25 mm layer thickness, Merck, Darmstadt, Germany) and RP-18 F_254S_ (Merck) plates; compounds were visualized by dipping plates into 20% (*v/v*) H_2_SO_4_ reagent (Aldrich, Milwaukee, WI , USA) and then heat-treated at 110 °C for 5–10 min. Silica gel (Merck 60A, 70–230 or 230–400 mesh ASTM), Sephadex LH-20 (Amersham Pharmacia Biotech), and Redi Sep-C18 (13 g, Teledyne Isco) were used for column chromatography. HPLC was performed using the Gilson Gastorr BG-34 degasser, Gilson 321 pump, Gilson UV/VIS-155 detector, with J’sphere ODS M-80 column (250 × 200 mm, i.d., 4 μm). All solvents used for the chromatographic separations were distilled before use.

### 3.2. Plant Meterial

The stem barks of *Ailanthus altissima* Swingle were purchased at humanherb Co. Gyeongsangbuk-do, Gyeong-san, Korea, in November 2011. A voucher specimen (No. 2012-AIAL01) has been deposited in the Lab of Natural Product Medicine, College of Pharmacy, Kyung Hee University, Seoul, Korea. 

### 3.3. Extraction and Isolation

The dried stem barks of *A. altissima* (14 kg) was extracted three times with 70% EtOH at room temperature, and then the solution was evaporated *in vacuo*. The EtOH extract (1.45 kg) was suspended in distilled water and then partitioned with *n*-hexane, EtOAc and BuOH, successively. A portion of the EtOAc-soluble layer (187 g) was subjected to silica gel column chromatography (CC) and eluted with a stepwise gradient of CH_2_Cl_2_–MeOH system (49:1 to 0:1, *v*/*v*) to afford 14 fractions (E1–E14). The fraction E4 (24.2 g) was chromatographed over silica gel (70–230 mesh) eluting with *n*-hexane–acetone (3:2 to 1:1, *v*/*v*) to produce 10 subfractions (E4-1–E4-10). Fraction E4-5 (1.85 g) and E4-6 (2.58 g) were further separated using a Sephadex LH-20 with CH_2_Cl_2_–MeOH mixture (1:1 *v*/*v*), yielding compounds **2** (9.4 mg), **7** (4.9 mg), **8** (11.0 mg), **9** (5.3 mg), and **10** (9.5 mg). The fraction E5 (16.04 g) was fractionated using the silica gel CC as stationary phase with a CH_2_Cl_2_–EtOAc mixture (1:1 to 3:7, *v*/*v*) as mobile phase to afford 10 subfractions (E5-1–E5-10). Compound **14** (9.7 mg) was isolated from fraction E5-5 (2.14 g) by Sephadex LH-20 CC using mixture of CH_2_Cl_2_–MeOH (1:1, *v*/*v*). The fraction E5-7 (900 mg) was successively fractionated using a Sephadex LH-20 with CH_2_Cl_2_–MeOH mixture (1:1, *v*/*v*) and flash chromatography system with Redi Sep-C18 (13 g, MeOH–H_2_O, 13:7 to 1:0, 7:13 to 13:7, *v*/*v*) to yield compounds **1** (3.6 mg) and **12** (10.6 mg). The fraction E6 (14.0 g) was separated by Silica gel (230–400 mesh) CC, using gradient mixtures of a CH_2_Cl_2_–acetone (4:1 to 1:1, *v*/*v*) as mobile phases, affording nine subfractions (E6-1–E6-9). Compounds **5** (0.7 mg) and **6** (1.4 mg) were purified from the subfraction E6-4 (1.03 g) using a Silica gel (230–400 mesh, *n*-hexane–EtOAc–MeOH, 3:2:1, *v*/*v*) and preparative HPLC (MeOH–H_2_O, 1:1 to 15:5, *v*/*v*), successively. The fraction E6-5 (1.25 g) was further fractionated using a silica gel (230–400 mesh) CC with CH_2_Cl_2_–EtOH–MeOH mixture (9:0.9:0.1 to 7:2.7:0.3, *v*/*v*), yielding compounds **4** (1.7 mg) and **5** (1.1 mg). The compound **13** (9.7 mg) was purified by recrystallization (in EtOAc) from the fraction E6-5-8 (273.1 mg). The fraction E6-7 (1.28 g) was purified further over a Sephadex LH-20 CC with CH_2_Cl_2_–MeOH mixture (1:1, *v*/*v*), yielding **15** (7.9 mg). Compound **11** (18.9 mg) was obtained from fraction E8 (190 mg) through Silica gel (230–400 mesh) CC (*n*-hexane–EtOAc–MeOH, 5:4:1 to 0:9:1, *v*/*v*). The BuOH–soluble layer (188 g) was chromatographed over silica gel CC as stationary phase with a CH_2_Cl_2_–MeOH–H_2_O mixture (9:1:0.1 to 7:2.8:0.2, *v*/*v*) as mobile phase to afford 15 pooled fractions (B1–B15). Fraction B8 (10.07 g) was subjected to a silica gel CC with CH_2_Cl_2_–MeOH mixture (17:3 to 0:1, *v*/*v*) to produce 6 subfractions (B8-1–B8-6). Compound **3** (1.1 mg) was purified from the subfraction B8-6 (600 mg) using a Sephadex LH-20 (CH_2_Cl_2_–MeOH, 1:1 to 0:1, *v*/*v*) and preparative HPLC (0.1% formic acid in MeCN-0.1% formic acid in H_2_O, 23:77, *v*/*v*), successively.

### 3.4. Spectral Data

*(R)-5-(1-Hydroxyethyl)-canthine-6-one (***1**): Yellow amorphous powder; ${\left[\alpha \right]}_{D}^{25}$ −13.68° (c 0.01, MeOH); UV (MeOH) λ_max_ (log ε) 229 (4.54), 258 (4.35), 298 (4.21), 359 (4.32), 375 (4.27) nm; IR (ATR) ν_max_ 3159, 2925, 1663, 1650, 1595, 1440, 1278, 1225, 1020, 746 cm^−1^; ^1^H- and ^13^C-NMR data, see Table 1; HR-DART-MS (positive mode) *m*/*z* 265.1006 [M + H]^+^; calcd. for C_16_H_13_N_2_O_2_, 265.0977).

### 3.5. Computational Methods

The conformational analysis was performed with Spartan’14 software package [33]and geometry optimizations were operated by the Gaussian 09 package [34]. TDDFT CD calculations for the optimized conformers were performed at the CAM-B3LYP/SVP level with a CPCM solvent model in MeCN. The CD spectra were generated by SpecDis 1.62 software [35].

### 3.6. Materials

Dulbecco’s modified Eagle’s minimum essential medium (DMEM), fetal bovine serum (FBS), penicillin, and streptomycin were obtained from Life Technologies Inc. (Grand Island, NY, USA). 3-(4,5-Dimethylthiazol-2-yl)-2,5-diphenyl-tetrazo-liumbromide (MTT), l-*N*^6^-(1-iminoethyl)lysine (l-NIL), LPS (Escherichia coli, serotype 0111:B4), and all other chemicals were purchased from Sigma Chemical Co.(St. Louis, MO, USA).

### 3.7. Cell Culture and Sample

RAW 264.7 macrophages were obtained from the Korea Cell Line Bank (Seoul, Korea). Cells were grown at 37 °C in DMEM medium supplemented with 10% FBS, penicillin sulfates in a humidified atmosphere of 5% CO_2_. Cells were pretreated with isolated compounds from *A. altissima* (7.5 μM/mL) or positive controls (l-NIL for iNOS inhibitor) for 1 h, and then stimulated with LPS (1 μg/mL) for the indicated time.

### 3.8. Cell Viability Assay

Cell viability studies were performed using the MTT (3-[4,5-dimethylthiazol-2yl]-2,5-dipheyl tetrazoliumbromide; Sigma-Aldrich, St. Louis, MO, USA) assay. Raw 264.7 cells were plated at a density of 0.9 × 10^5^ cells/mL in 96-well. Cells were pretreated with isolated compounds from *A. altissima* (50 μM) for 1 h and then stimulated with LPS (1 μg/mL) for 24 h. Then 50 μL of MTT solution (5 mg/mL in PBS) was added to the medium and the cells were incubated at 37 °C for 4 h. The MTT-containing medium was removed and the cells were solubilized in DMSO (100 μL) for 10 min. The optical density at 540 nm was determined using a microplate spectrophotometer (Molecular Devices Inc., Sunnyvale, CA, USA) to determine the cell viability.

### 3.9. Measurment of Nitric Oxide Production

RAW264.7 cells were plated at 2 × 10^5^ cells/well in 60 mm dishes and incubated with or without LPS (1 μg/mL) in the absence or presence of indicate concentration of the samples for 24 h. The nitrite which accumulated in culture medium was measured as an indicator of NO production according to the Griess reagent. The culture supernatant (100 μL) was mixed with 100 μL of Griess reagent [equal volumes of 1% (*w*/*v*) sulfanilamide in 5% (*v*/*v*) phosphoric acid and 0.1% (*w*/*v*) naphthyl ethylenediamine-HCl] for 10 min, and then the absorbance at 540 nm was measured in a microplate reader. Fresh culture medium was used as the blank in all experiments. The amount of nitrite in the samples was determined with reference to a sodium nitrite standard curve.

## 4. Conclusions

The new canthin-6-one alkaloid (**1**) and 14 known compounds (**2**–**15**) were isolated from the stem bark of *A. altissima.* The structure of the new compound (**1**) was elucidated by its physical and spectroscopic data, and its absolute configuration was determined by comparison of its experimental and calculated ECD spectra. Compounds **3**–**9** and **12** were isolated from this plant for the first time. The isolates were screened for inhibitory activity against LPS-induced NO production in RAW 264.7 cells. Compounds **1**, **2**, **4**, **5**, **9** and **11** exhibited a significant inhibitory activity, with IC_50_ values in the range of 5.92 ± 0.9 to 15.09 ± 1.8 μM. Thus, the active isolates seem to be worthy of additional biological tests to more fully evaluate the plant’s potential as a therapeutic agent for anti-inflammatory diseases with an excess production of NO.

## Figures and Tables

**Table 1:** ^1^H-NMR (500 MHz), ^13^C-NMR (125 MHz), COSY, HMBC spectroscopic data for compound **1** in CD_3_OD.

Position	δ_H_ Mult., (*J* in Hz)	δ_C_	COSY	HMBC
1	8.16 d (5.0)	117.6	H-2	H-2
2	8.77 d (5.0)	146.8	H-1	H-1
4	8.18 d (1.0)	133.5		H-17
5		147.8		H-17, H-18
6		160.2		H-4, H-17
8	8.60 d (8.0)	118.0	H-9	H-10
9	7.74 dt (8.0, 1.0)	132.1	H-8, H-10	
10	7.58 dt (8.0, 1.0)	127.1	H-9, H-11	
11	8.26 d (8.0)	124.4	H-10	H-9
12		126.1		H-1, H-8, H-10
13		140.9		H-9, H-11
14		131.7		H-2, H-11
15		132.5		H-1, H-4
16		137.0		H-2
17	5.16 dq (6.5, 1.0)	66.1	H-18	H-4, H-18
18	1.57 d (6.5)	23.4	H-17	H-17

**Table 2:** Inhibitory effects of **1**–**15** isolated from the stem bark of *A. altissima* on nitric oxide production in LPS-induced RAW 264.7 cells.

Compound	IC_50_ (μM) *	Compound	IC_50_ (μM) *	Compound	IC_50_ (μM) *
**1**	15.09 ± 1.8	**6**	>50	**11**	10.69 ± 0.4
**2**	9.09 ± 0.34	**7**	>50	**12**	>50
**3**	ND **	**8**	>50	**13**	>50
**4**	7.73 ± 0.3	**9**	5.92 ± 0.9	**14**	>50
**5**	12.01 ± 0.1	**10**	>50	**15**	>50

* IC_50_ value is defined as the concentration that results in a 50% decreased production of nitric oxide. The values represent the means of the results from three independent experiments with similar patterns. l-*N*^6^-(1-iminoethyl)lysine (l-NIL) was used as assay positive control for NO production (IC_50_ value: 15.8 μM). ** ND: not determined due to a limited amount of the sample.

## Supplementary Materials

Supplementary materials can be accessed at: http://www.mdpi.com/1420-3049/21/5/642/s1.
