# Supplementary material for: A New Canthinone-Type Alkaloid Isolated from Ailanthus altissima Swingle
Source: Molecules. 2016 May 16;21(5):642. doi: 10.3390/molecules21050642 (PMC6273678; doi:10.3390/molecules21050642)
Supplement: Supplementary file 1 [file molecules-21-00642-s001.pdf]

Supplementary Materials: A New Canthinone-Type Alkaloid Isolated from *Ailanthus altissima* Swingle

Hye Mi Kim, Jin Su Lee, Jurdas Sezirahiga, Jaeyoung Kwon, Miran Jeong, Dongho Lee, Jung-Hye Choi and Dae Sik Jang

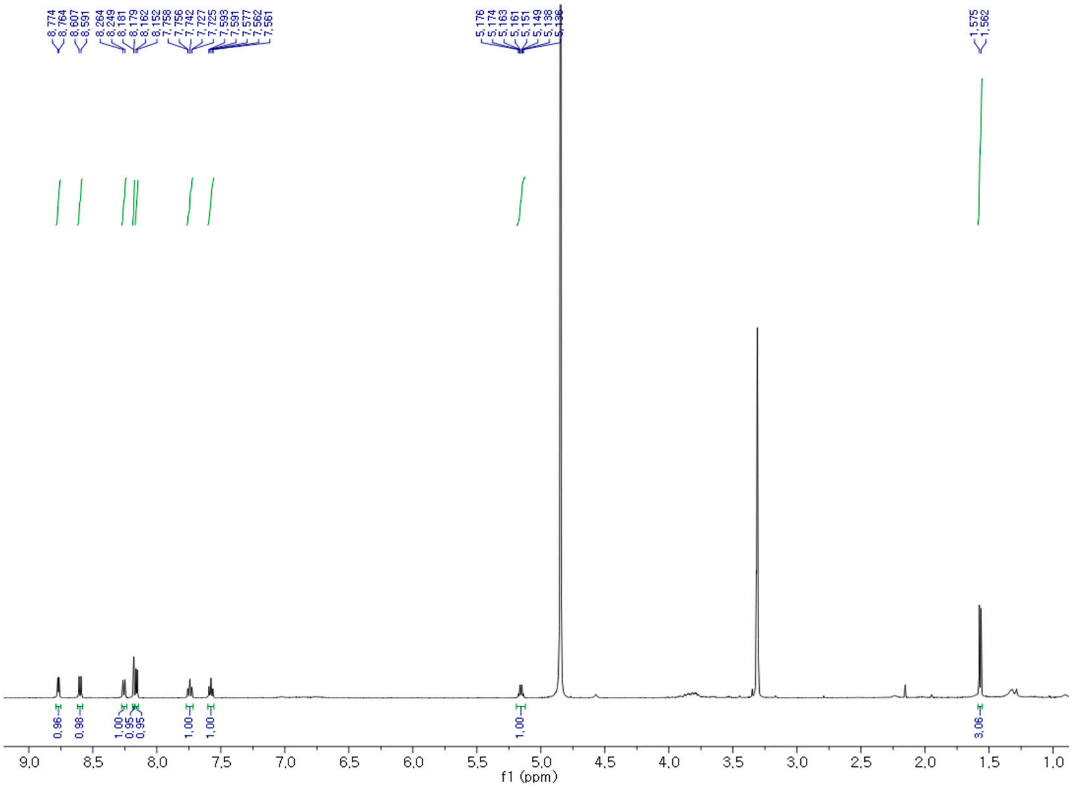

Figure S1. The ^{1}H-NMR spectrum of compound **1** (CD_3OD, 500MHz).

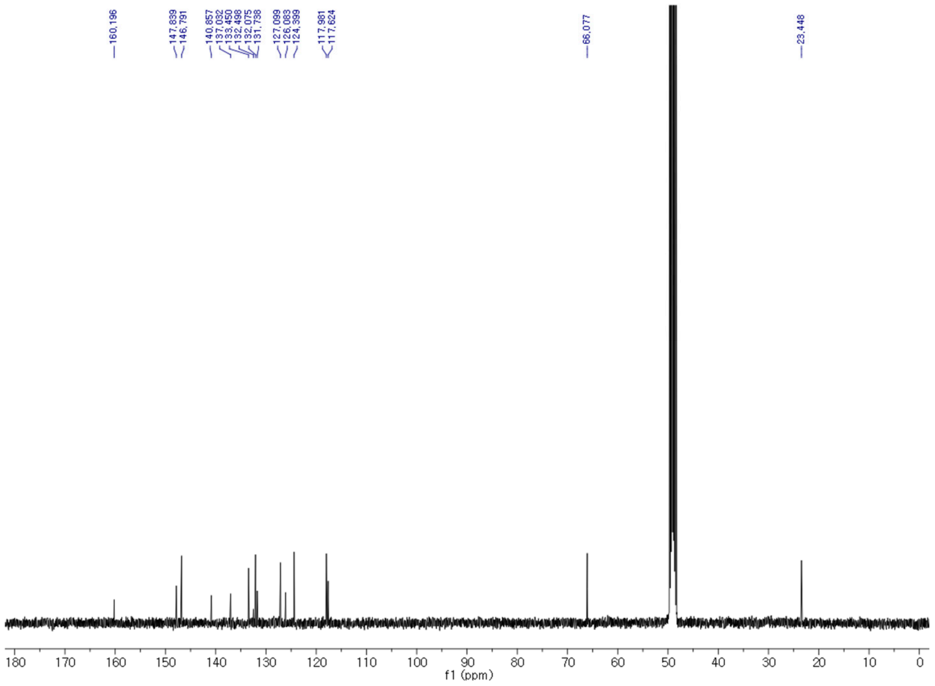

Figure S2. The ^{13}C-NMR spectrum of compound **1** (CD_3OD, 125MHz).

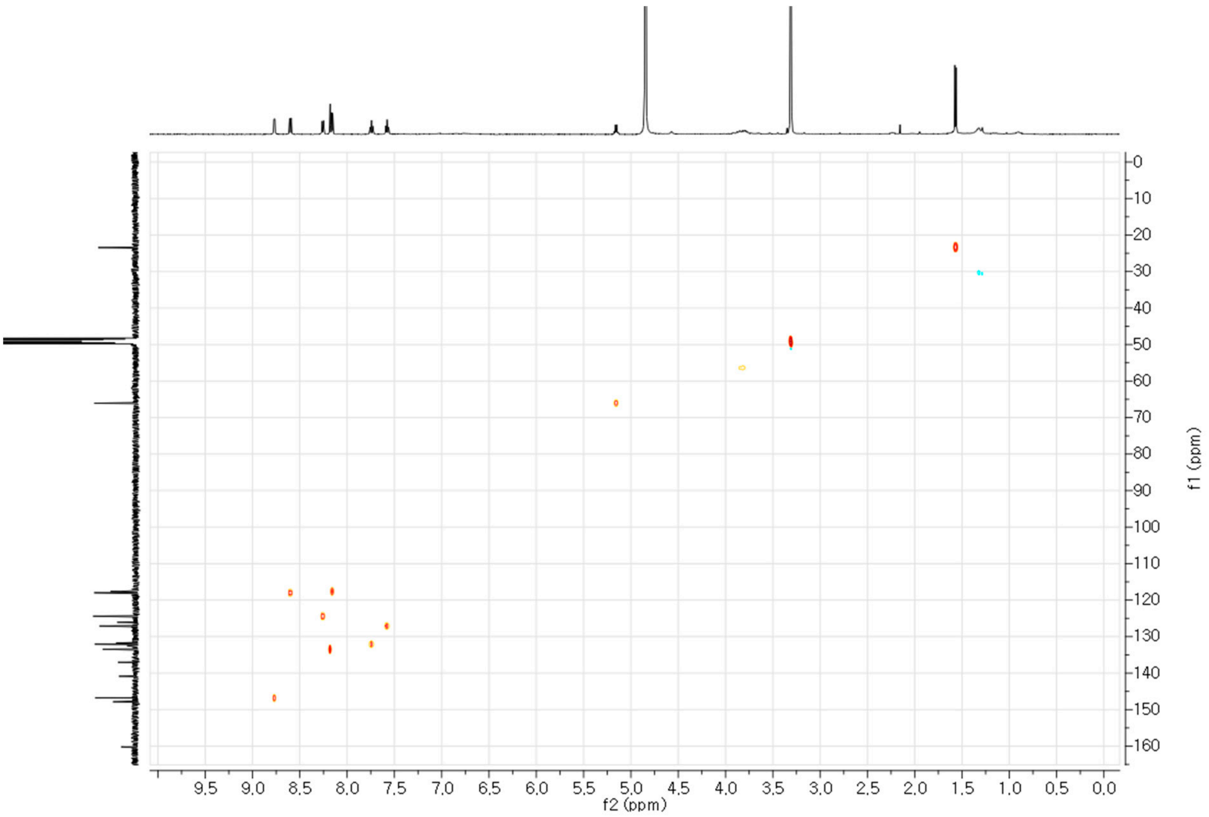

Figure S3. The 2D NMR (HMQC) spectrum of compound **1** (CD_3OD).

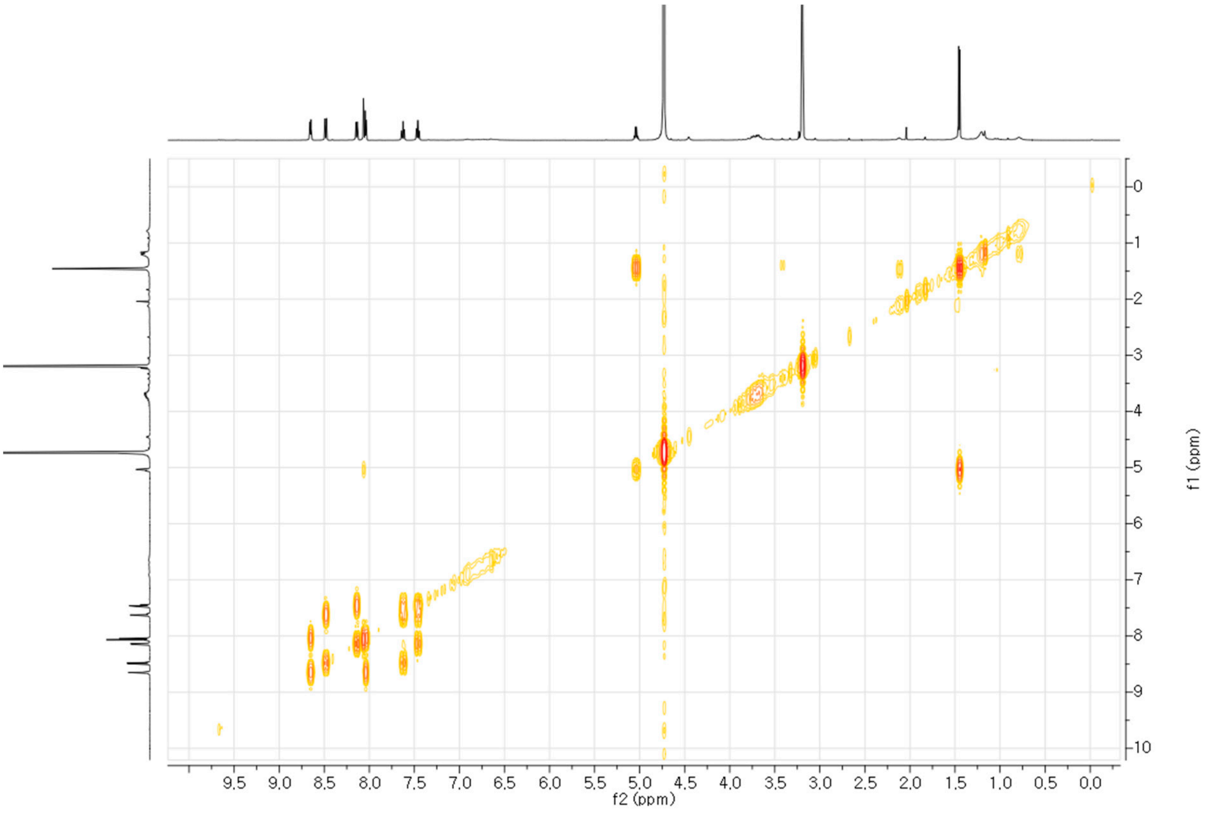

Figure S4. The 2D NMR (^1H-^1H COSY) spectrum of compound **1** (CD_3OD).

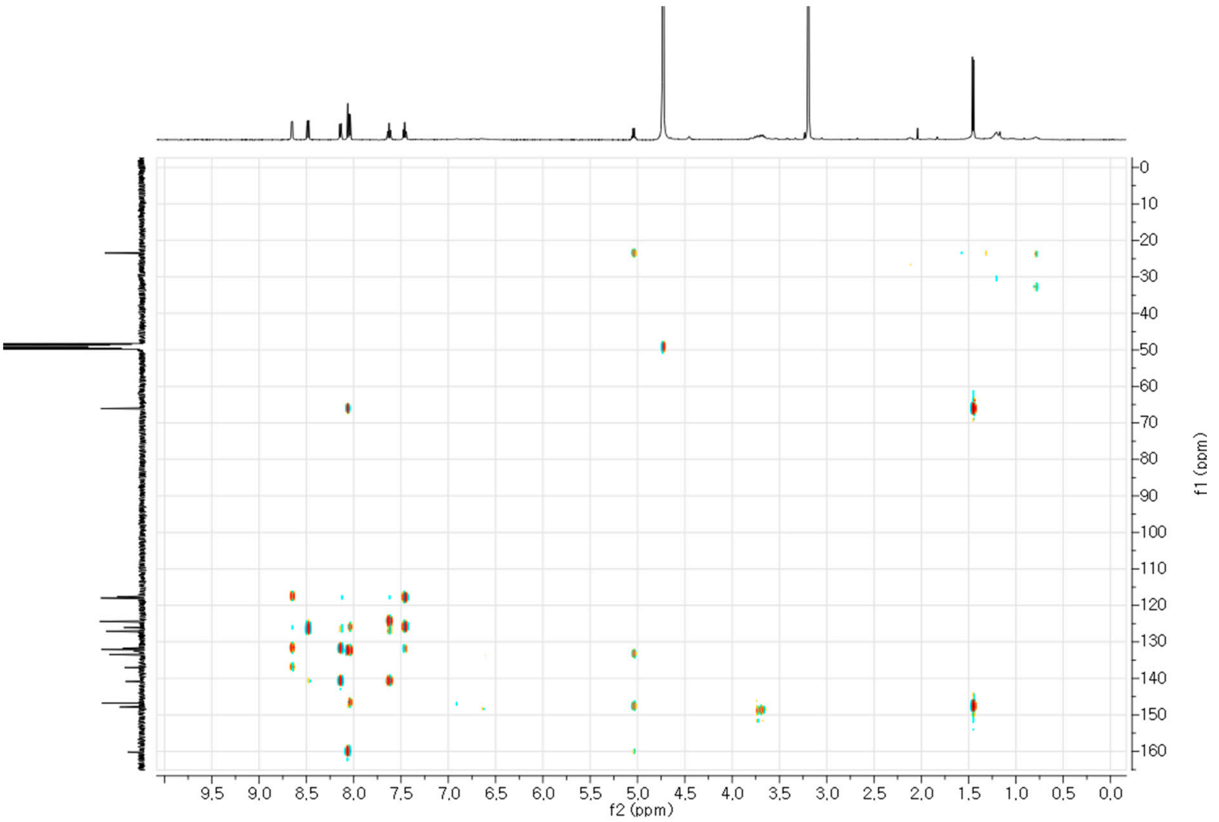

Figure S5. The 2D NMR (HMBC) spectrum of compound **1** (CD_3OD).

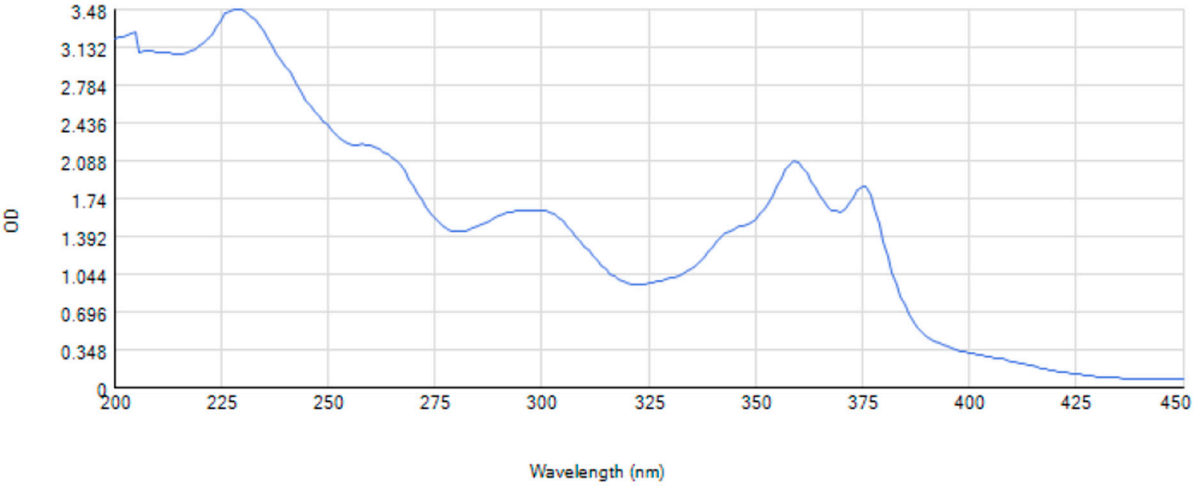

Figure S6. The UV spectrum of compound **1** (MeOH).

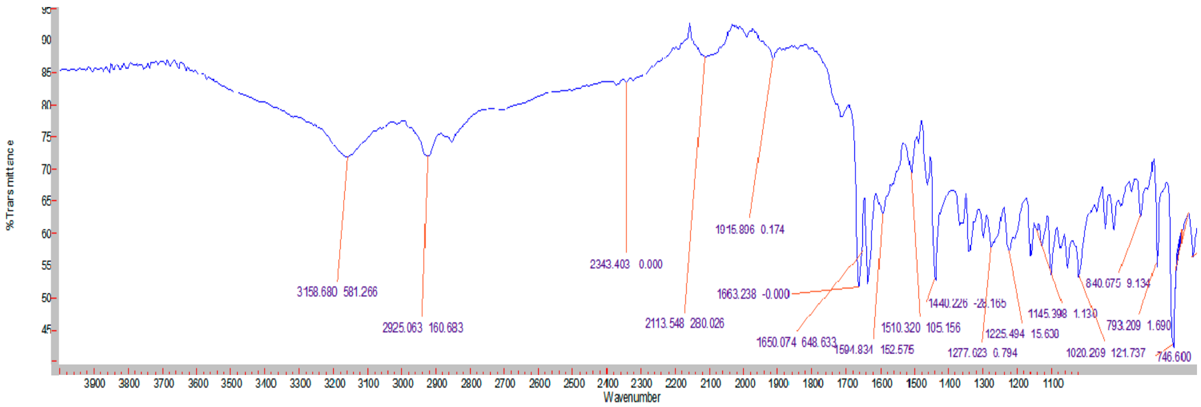

Figure S7. The IR spectrum of compound **1** (MeOH).

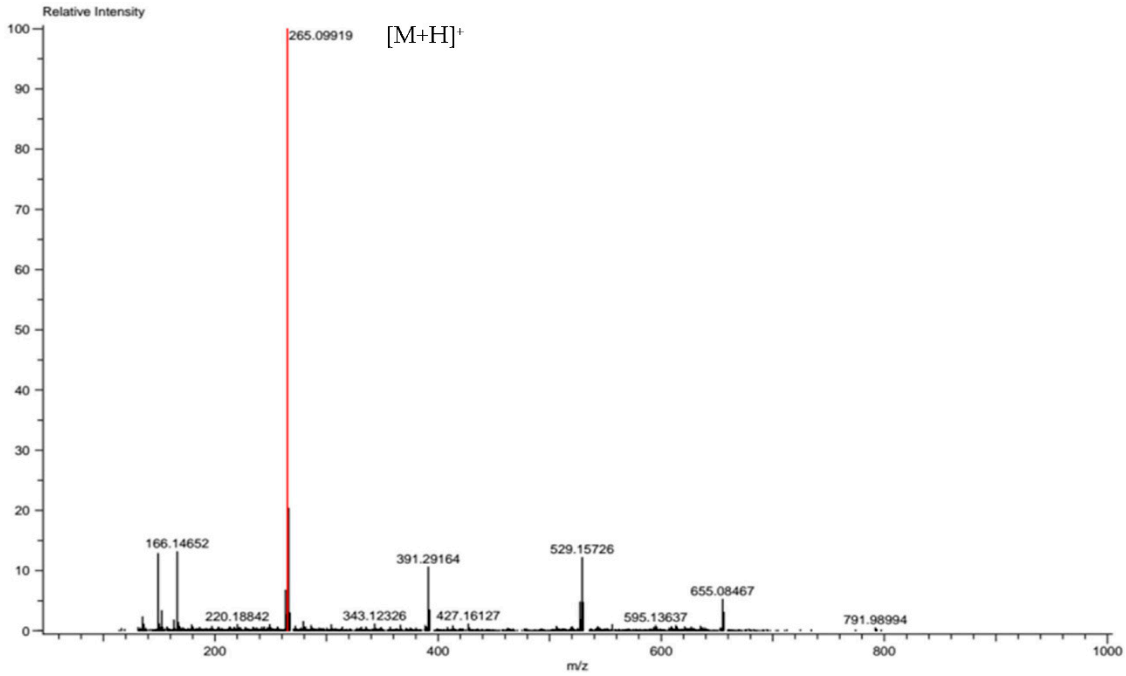

Figure S8. The HR-DART-MS spectrum of compound **1** (*m*/*z* 265.0992 [M + H]$^+$; calcd for $C_{16}H_{13}N_2O_2$, 265.0977).
